# Turnover of the mTOR inhibitor, DEPTOR, and downstream AKT phosphorylation in multiple myeloma cells, is dependent on ERK1-mediated phosphorylation

**DOI:** 10.1016/j.jbc.2022.101750

**Published:** 2022-02-23

**Authors:** Mario Vega, Yu Chen, Yijiang Shi, Joseph Gera, Alan Lichtenstein

**Affiliations:** 1 Hematology-Oncology, Greater Los Angeles VA-UCLA Healthcare Center, UCLA and Jonsson Comprehensive Cancer Center, UCLA, Los Angeles, California, USA; 2 Molecular Instrumentation Center, UCLA and Jonsson Comprehensive Cancer Center, UCLA, Los Angeles, California, USA

**Keywords:** multiple myeloma, DEPTOR, mTOR, ERK, AKT, β-TrCP, beta-transducin repeat–containing protein, ACN, acetonitrile, AKT, serine/threonine-specific protein kinase, CK1α, casein kinase 1α, DEP, Disheveled, Egl-10, and Pleckstrin, DEPTOR, Disheveled, Egl-10, and Pleckstrin domain–containing mechanistic target of rapamycin–interacting protein, ERK1, extracellular signal–regulated kinase 1, EV, empty vector, FDR, false discovery rate, HDM2, human and/or murine double minute-2 protein, Ig, immunoglobulin, IRS-1, insulin receptor substrate 1, LC, liquid chromatography, MAF, musculoaponeurotic fibrosarcoma, MM, multiple myeloma, MS, mass spectrometry, mTOR, mechanistic target of rapamycin, OTUB1, ovarian tumor deubiquitinase, ubiquitin aldehyde binding 1, PD, PD98059 ERK inhibitor, PTM, post-translational modification, RAS, rat sarcoma virus, S235, serine 235, S–A, serine-to-alanine, S–Asp, serine-to-aspartate, TORC1, TOR kinase complex 1, USP-7, ubiquitin-specific protease 7

## Abstract

DEPTOR is a 48 kDa protein upregulated in multiple myeloma (MM) cells. DEPTOR inhibits mTOR and, by repressing a negative feedback loop, promotes AKT activation. We previously identified a compound that binds to DEPTOR in MM cells and induces its proteasomal degradation. To identify the mechanism of degradation, here, we screened for drug-induced posttranslational modifications and identified reduced phosphorylation of DEPTOR on serine 235 (S235). We show that an S235 phosphomimetic DEPTOR mutant was resistant to degradation, confirming the importance of this posttranslational modification. In addition, a DEPTOR mutant with a serine-to-alanine substitution at S235 could only be expressed upon concurrent proteasome inhibition. Thus, S235 phosphorylation regulates DEPTOR stability. Screening the DEPTOR interactome identified that the association of USP-7 deubiquitinase with DEPTOR was dependent upon S235 phosphorylation. Inhibition of USP-7 activity resulted in DEPTOR polyubiquitination and degradation. A scansite search suggested that ERK1 may be responsible for S235 phosphorylation, which was confirmed through the use of inhibitors, ERK1 knockdown, and an *in vitro* kinase assay. Inhibition of ERK1 also downregulated AKT phosphorylation. To test if DEPTOR phosphorylation mediated this crosstalk, MM cells were transfected with WT or phosphomimetic DEPTOR and exposed to ERK inhibitors. Although WT DEPTOR had no effect on the inhibition of AKT phosphorylation, the phosphomimetic DEPTOR prevented inhibition. These results indicate that ERK1 maintains AKT activity in MM cells via phosphorylation of DEPTOR. We propose that DEPTOR-dependent crosstalk provides MM cells with a viability-promoting signal (through AKT) when proliferation is stimulated (through ERK).

Disheveled, Egl-10, and Pleckstrin (DEP) domain–containing mechanistic target of rapamycin (mTOR)–interacting protein (DEPTOR) is a 48 kDa protein that binds to the mTOR and downregulates its kinase activity within TOR kinase complex 1 (TORC1) and TOR kinase complex 2 (1). DEPTOR contains two tandem DEP domains and a C-terminal PDZ domain. The function of the DEP domains is unknown, but the PDZ domain mediates protein–protein interactions, and DEPTOR binds mTOR *via* its PDZ domain. By repressing a negative feedback loop, DEPTOR-induced inhibition of TORC1 results in activation of the PI3K–AKT (a serine/threonine-specific protein kinase) pathway (1). As an mTOR inhibitor, it is not surprising that DEPTOR expression is low in most cancer subtypes. However, expression is particularly elevated in multiple myeloma (MM) tumor cells (1). High MM-specific DEPTOR expression has been explained as a mechanism to restrain cap-dependent translation and prevent endoplasmic reticulum stress (by inhibiting TORC1) while, at the same time, maintaining activated PI3K–AKT (by repressing the negative feedback loop) to prevent apoptosis. It is notable that MM tumor cells are especially sensitive to endoplasmic reticulum stress because of overactive immunoglobulin (Ig) synthesis (2).

DEPTOR expression can be regulated at the transcriptional level. Indeed, the highest levels of DEPTOR expression in MM occur within the subtype that contains Ig heavy locus translocations resulting in heightened activity of the transcription factors C-musculoaponeurotic fibrosarcoma (MAF) and v-maf avian MAF oncogene homolog B (1). Furthermore, knockdown of MAF in MM cell lines inhibits DEPTOR expression (1). However, DEPTOR levels are also controlled by post-translational mechanisms. Three separate groups (3, 4, 5) have described a pathway whereby DEPTOR phosphorylation results in an association with the beta-transducin repeat–containing protein (β-TrCP) E3 ubiquitin ligase with subsequent ubiquitination and proteasomal degradation. Binding to β-TrCP occurs *via* phosphorylated residues in a conserved degron sequence between amino acids 286 and 291. Phosphorylation events seem to be mediated by a synergistic interaction between the mTOR kinase and casein kinase 1α (CK1α), although there may also be a role for the S6 kinase 1, downstream of mTOR, and the ribosomal protein S6 kinase 1 (5). These data suggest a feedforward loop where mTOR activity becomes amplified by promoting phosphorylation and degradation of its negative regulator. However, other post-translational events may also regulate DEPTOR's proteasomal degradation. For example, KDM4A, a demethylase controlling protein methylation, enhances DEPTOR stability by repressing its ubiquitination (6).

In previous work (7, 8, 9), we have described small inhibitors that bind to the PDZ domain of DEPTOR, prevent DEPTOR/mTOR association in MM cells, induce acute activation of mTOR, and MM tumor cell apoptosis. Most interesting, our second-generation inhibitor, termed drug 3g, not only prevented DEPTOR/mTOR association but also induced rapid proteasomal degradation of DEPTOR, adding to the anti-MM effect (9). Drug-induced DEPTOR destabilization was not because of an activation of mTOR or degron phosphorylation (9).

The aforementioned data indicated a novel mechanism of DEPTOR degradation, which is the subject of this report. Our results show that drug 3g induces a dephosphorylation of DEPTOR on serine 235 (S235), which results in release of the ubiquitin-specific protease 7 (USP-7) deubiquitinase. Polyubiquitination ensues with proteasomal degradation. The critical phosphorylation state of S235 is mediated by the extracellular signal–regulated kinase 1 (ERK1). Furthermore, our results suggest that the level of S235 phosphorylation is also a physiologic regulator of DEPTOR turnover in the absence of drug 3g and that DEPTOR S235 phosphorylation plays a key role in the crosstalk between ERK and AKT in MM cells.

## Results

### Drug 3g causes dephosphorylation of DEPTOR at S235

**Figure 1:**
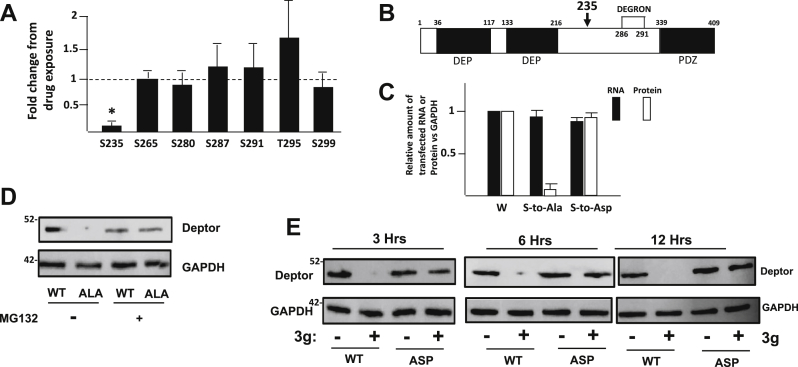
**Critical role of serine 235 phosphorylation.***A*, fold change in DEPTOR phosphorylation on several residues induced by drug 3g (mean ± SD, N = 4); ∗ represents significant change, *p* < 0.05, from control (untreated). *B*, schematic of DEPTOR structure. *C*, relative RNA and protein expression (*versus* GAPDH) of different transfected DEPTOR genes in 8226 cells; WT, serine-to-alanine mutant, or serine-to-aspartate mutant; data are mean ± SD, n = 3. *D*, immunoblot assay for expression of WT *versus* serine-to-alanine mutant 6 h after transfection when cultured with or without MG-132. *E*, immunoblot assay for expression of WT *versus* serine-to-aspartate mutant DEPTOR after exposure to drug 3g (1 μM) for 3, 6, or 12 h in 8226 cells. DEPTOR, Disheveled, Egl-10, and Pleckstrin domain–containing mechanistic target of rapamycin–interacting protein.

The 8226 MM cell line contains high levels of DEPTOR because of MAF dysregulation (1, 7) and is very sensitive to drug 3g-induced DEPTOR degradation (9). Thus, we exposed 8226 cells to drug 3g (1 μM) + the proteasome inhibitor MG-132 (to prevent DEPTOR degradation) for 4 h and performed a mass spectrometry (MS) analysis to identify post-translational alterations. MS identified multiple phosphorylation sites (S235, S265, S280, S287, S291, T295, and S299) with total DEPTOR sequence coverage of 94%. When compared with cells exposed to MG-132 alone, there were no significant changes in the phosphorylation status of residues S287 and S291, located within the β-TrCP degron (Fig. 1*A*). Gao *et al.* (4) reported that mTOR also phosphorylates DEPTOR on residues S299 and T295, surrounding the degron, which primes for subsequent phosphorylation of S286 and S287. Although drug 3g did increase T295 phosphorylation, this did not reach statistical significance. S299 phosphorylation was not altered. The absence of enhanced phosphorylation of the DEGRON and its surrounding residues is consistent with our previous work (9), demonstrating that drug 3g-induced DEPTOR degradation is not because of heightened mTOR-mediated phosphorylation. On the other hand, although drug 3g rapidly prevents binding of mTOR to DEPTOR (9), and thus, might be expected to actually decrease mTOR-dependent degron phosphorylation, a 4 h exposure may not have been sufficient to identify such a decrease. In contrast to an absence of drug effect on these residues, a remarkable drug-induced decrease in phosphorylation was identified at S235 (Fig. 1*A*). S235 is located between the C-terminal DEP domain and the degron (Fig. 1*B*). Spectra of S235 phosphorylation specifically are shown in Fig. S1, and accessing spectra for all phosphopeptide identification can be found in the “Data availability” section under “Experimental procedures” section. No other significant alterations of post-translational modifications (PTMs) induced by drug 3g were identified. All phosphopeptide identification can also be accessed through the site shown in the “Data availability” section.

To test the relevance of diminished S235 phosphorylation, we generated DEPTOR mutants that would resist S235 phosphorylation (serine-to-alanine [S–A]) or present a phosphomimetic signal (serine-to-aspartate [S–Asp]) at residue 235. FLAG-tagged constructs were transfected into MM cells, and immunoblot was performed for tagged DEPTOR expression. Although both mutants were normally transcribed, there was no protein expression of the S–A mutant identified (Fig. 1*C*), and simultaneous blocking of proteasome function with MG-132 allowed normal S–A protein expression (Fig. 1*D*). Thus, the absence of S235 phosphorylation destabilizes DEPTOR protein resulting in proteasomal degradation. As expected, when the S–A mutant was protected by MG-132, exposure to drug 3g had no effect on expression (Fig. S2*A*). To test if decreased phosphorylation at S235 mediates drug-induced DEPTOR degradation, 8226 cells were transfected with either tagged WT or the phosphomimetic S–Asp mutant and treated in the presence or the absence of drug 3g for 3, 6, or 12 h. Drug 3g (1 μM) induced WT DEPTOR degradation rapidly by 3 h (Fig. 1*E*). This rapid kinetics is comparable to drug-induced degradation of endogenous DEPTOR in sensitive MM cells (9). In contrast, drug 3g was incapable of destabilizing and degrading the S–Asp mutant even with exposures up to 12 h (Fig. 1*E*). These results confirm the relevance of S235 dephosphorylation in drug-induced DEPTOR fragmentation. In addition, the results with the S–A mutant shown in Figure 1*D* also indicate S235 phosphorylation physiologically regulates DEPTOR stability in the absence of drug 3g. There was only a slight prolongation of the *T*_1/2_ of the aspartate mutant *versus* WT DEPTOR in the absence of drug 3g (Fig. S2*B*). This was probably explained by the finding that, in the 8226 cell line, WT DEPTOR is constitutively phosphorylated at S235 (see later) so there is little difference in *T*_1/2_ *versus* the phosphomimetic mutant.

### Drug 3g decreases association of DEPTOR to the USP-7 deubiquitinase

**Figure 2:**
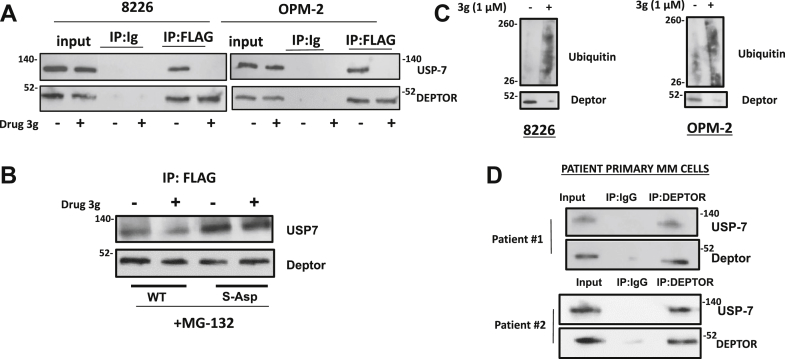
**Association of DEPTOR with USP-7.***A*, coimmunoprecipitation (co-IP) in 8226 or OPM-2 MM cells after pulling down FLAG-DEPTOR and immunoblotting for USP-7; IPs were performed after culture for 4 h with or without drug 3g (1 μM). *B*, 8226 MM cells transfected with FLAG-tagged WT or S–Asp mutant and after 4 h with or without drug 3g, co-IP assay performed for USP-7 binding. *C*, 8226 or OPM-2 MM cells treated with or without drug 3g for 4 h and then assayed for ubiquitination of DEPTOR. *D*, isolated primary MM cells from two patients where endogenous DEPTOR was immunoprecipitated and blotted for associated USP-7. DEPTOR, Disheveled, Egl-10, and Pleckstrin domain–containing mechanistic target of rapamycin–interacting protein; MM, multiple myeloma; USP-7, ubiquitin specific protease 7.

We next performed MS analysis to identify any drug-induced alteration of proteins bound to DEPTOR. Tagged DEPTOR was immunoprecipitated after a 4 h exposure to drug 3g (1 μM) + MG-132 and compared with cells treated with MG-132 alone. Nonspecific binders were identified and discarded by use of IgG instead of anti-DEPTOR immunoprecipitating antibody. In the absence of drug 3g, 242 distinct binding proteins were identified by MS. Following exposure to drug 3g, there were 238 distinct DEPTOR binders identified. Combining the proteins bound to DEPTOR in the basal condition (*i.e.*, no drug 3g) with those identified after drug 3g treatment yielded a dataset of 301 proteins. From these 301 separate proteins, the DEPTOR binding of 49 proteins significantly decreased (two-tailed *p* value <0.05, Table S1), and the binding of 25 proteins significantly increased following drug treatment (Table S2). For all other protein-binder identification, see the site identified in the “Data availability” section. As expected from our previous work (9), binding to mTOR was significantly decreased by drug 3g (treated/nontreated ratio = 0.28 ± 0.05, mean ± SD, *single arrow* in Table S1). Also as expected, drug 3g exposure resulted in a significant increase in binding of a number of proteasomal subunits (*arrows* in Table S2), consistent with the overall effect of sensitizing DEPTOR for proteasome degradation. Of all 301 proteins, the USP-7 deubiquitinase demonstrated the most significant decrease in DEPTOR binding following drug exposure (treated/nontreated ratio = 0.05 ± 0.01, *double arrow* in Table S1). Confirmation of the MS results is shown in Figure 2*A*, where either 8226 or OPM-2 MM cells, expressing tagged DEPTOR, were treated in the presence or the absence of drug 3g for 4 h in the presence of MG-132, following which DEPTOR was immunoprecipitated and immunoblotted for bound USP-7. OPM-2 is an additional MM cell line with abnormally upregulated DEPTOR expression. As shown, drug 3g significantly decreased DEPTOR/USP-7 binding after this short exposure in both cell lines. We next tested if the drug-induced dephosphorylation at S235 was critical for USP-7 binding by transfecting either WT FLAG-DEPTOR or the S–Asp phosphomimetic mutant, treating in the presence or the absence of drug 3g for 4 h in the presence of MG-132 and testing for bound USP-7 on anti-FLAG DEPTOR immunoprecipitates. As shown in Figure 2*B*, drug 3g effectively caused decreased binding of USP-7 to WT DEPTOR but had no effect on binding to the phosphomimetic mutant. Depressed binding of and action of a deubiquitinase should lead to enhanced DEPTOR ubiquitination. This was tested in drug-treated 8226 and OPM-2 cells. As shown in Figure 2*C*, enhanced ubiquitination of endogenous DEPTOR was evident in both cell lines treated with drug 3g in association with loss of expression.

We were also fortunate to obtain sufficient primary bone marrow MM cells from two newly diagnosed patients to perform coimmunoprecipitation by immunoprecipitating endogenous DEPTOR. Figure 2*D* demonstrates that USP-7 is constitutively bound to DEPTOR in these patient samples.

### Targeting USP-7 results in DEPTOR destabilization

**Figure 3:**
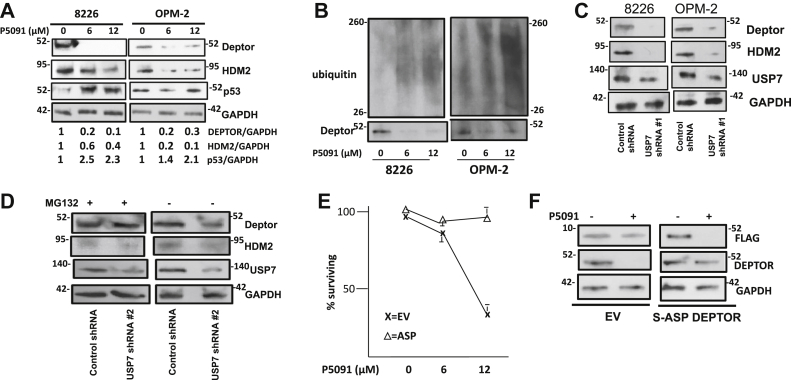
**Targeting USP-7 induced DEPTOR ubiquitination and degradation.***A*, 8226 or OPM-2 MM cells exposed to the P5091 USP-7 inhibitor for 4 h followed by immunoblot assay; below gels are relative expression of DEPTOR, HDM2, and p53 from three experiments (mean values). *B*, MM cell lines exposed to P5091 for 4 h followed by assay for DEPTOR polyubiquitination. *C*, MM cell lines transfected with control shRNA (targeting scrambled sequence) or USP-7 shRNA 1 and 14 h later assayed by immunoblot. *D*, 8226 cells transfected with control or a second different USP-7. shRNA (2) and then exposed in the presence or the absence of MG-132 for 12 h followed by immunoblot assay. *E*, MTT assay of 8226 cells transfected with either EV or S–Asp mutant and challenged with P5091 for 48 h. Data are percent of survival, mean ± SD, n = 4. *F*, EV- or S–Asp-transfected MM cells exposed to P5091 (12 μM for 48 h) following which immunoblot assay was performed. DEPTOR, Disheveled, Egl-10, and Pleckstrin domain–containing mechanistic target of rapamycin–interacting protein; EV, empty vector; HDM2, human and/or murine double minute-2 protein; MM, multiple myeloma; MTT, 3-(4,5-dimethylthiazol-2-yl)-2,5 diphenyl tetrazolium bromide; S–Asp, serine-to-aspartate; USP-7, ubiquitin-specific protease 7.

To test if USP-7 binding regulated DEPTOR stability, we first utilized a novel USP-7 inhibitor, P5091 (Fig. 3*A*). This inhibitor has shown promise as an anti-MM drug, and one of its proposed mechanisms is that USP-7 paralysis results in an enhanced ubiquitination and fragmentation of human and/or murine double minute-2 protein (HDM2) with subsequent p53 activation, which is thought to mediate MM cell death (10). We, thus, treated 8226 or OPM-2 MM cells with 0, 6, or 12 μM of P5091 for 4 h. The ID_50_ of P5091 for the 8226 cell line after 48 h of exposure is approximately 12.5 μM (10). At our time point of 4 h, there was no discernible toxicity to either 8226 or OPM-2 cells. Figure 3*A* is a representative experiment demonstrating that, as expected, P5091 caused decreased HDM2 expression and a corresponding increased p53 expression (semiquantifications of protein expression by densitometry from three independent experiments are shown below the gels as means). Also, targeting USP-7 resulted in loss of DEPTOR expression in both MM cell lines (20 ± 7% of control at 6 μM P5091 and 12 ± 5% at 12 μM for 8226 cells and 23 ± 6% at 6 μM and 27 ± 5% at 12 μM for OPM-2 cells, mean ± SD, n = 3). This was associated with enhanced ubiquitination of DEPTOR (Fig. 3*B*). Comparable results were seen when USP-7 was targeted genetically by two separate shRNAs in 8226 or OPM-2 cells (Fig. 3, *C* and *D*). As shown, the silencing of USP-7 was associated with decreased expression of HDM2 and DEPTOR (Fig. 3*C*) in both MM cell lines. A second independent shRNA was used in 8226 cells (Fig. 3*D*) to further support the negative effect on DEPTOR expression when USP-7 is silenced. In addition, Figure 3*D* demonstrates that this negative effect is reversed by MG-132. Thus, targeting USP-7 pharmacologically and genetically results in DEPTOR ubiquitination and degradation and further supports the notion that loss of DEPTOR–USP-7 binding is the key molecular alteration that mediates drug 3g-induced DEPTOR degradation.

As described previously, the P5091 USP-7 inhibitor is selectively cytotoxic to MM cells (10), possibly because of activated p53. However, we considered the possibility that the associated degradation of DEPTOR in P5091-treated MM cells could contribute to cytotoxicity. To test this, the FLAG-tagged phosphomimetic DEPTOR (S–Asp mutant) was ectopically expressed in 8226 cells (*versus* a FLAG-tagged empty vector [EV]) followed by challenge with P5091. Results shown in Figure 3*E* suggest that DEPTOR overexpression affords some protection against P5091, specifically identified at the 12 μM dose. Although P5091, by inhibiting USP-7, would be expected to induce some degradation of the S–Asp–transfected DEPTOR protein, there may have been a sufficient level still remaining to protect against cytotoxicity. An immunoblot assay (Fig. 3*F*) performed after 48 h exposure to 12 μM of P5091 suggests this possible explanation. As shown, the P5091 USP-7 inhibitor induced degradation of the FLAG-tagged DEPTOR while, as expected, having no effect on expression of the FLAG-tagged EV. However, following exposure to P5091, there was still some DEPTOR expression remaining in DEPTOR-transfected cells compared with EV-transfected cells (the latter cells demonstrating complete loss of expression of endogenous DEPTOR).

### Role of the ERK in DEPTOR turnover

**Figure 4:**
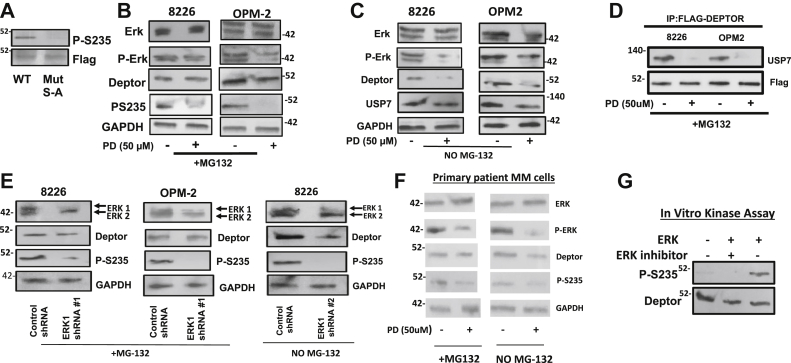
**S235 phosphorylation is mediated by ERK1.***A*, WT or serine-to-alanine (S–A) mutant DEPTOR was expressed and immunoprecipitated from 8226 cells. The immunoprecipitate was. immunoblotted with 235 phosphospecific antibody. *B*, MM cells treated in the presence or the absence of PD98059 (PD) for 4 h in the presence of MG-132 followed by immunoblot assay. *C*, similar experimental design as in (*B*) except no MG-132 present. *D*, MM cells transfected with FLAG-DEPTOR and then treated in the presence or the absence of PD98059 (4 h) followed by co-IP assay for USP-7 binding to DEPTOR. *E*, MM cells exposed to control shRNA (targeting scrambled sequence) or ERK1 shRNA 1 or 2 and then cultured in the presence or the absence of MG-132 (for 24 h) followed by immunoblot assay. *F*, isolated primary MM cells exposed in the presence or the absence of PD 98059 and with/without MG-132 for 4 h, followed by immunoblot assay. *G*, *in vitro* kinase assay testing S235 phosphorylation of recombinant DEPTOR ± recombinant ERK. co-IP, coimmunoprecipitation; DEPTOR, Disheveled, Egl-10, and Pleckstrin domain–containing mechanistic target of rapamycin–interacting protein; ERK1, extracellular signal–regulated kinase 1; MM, multiple myeloma; S235, serine 235; USP-7, ubiquitin-specific protease 7.

A scansite search for kinase consensus sites identified ERK1 as a potential mediator of S235 phosphorylation. In addition, a previous study (5) demonstrated that ERK1 can bind DEPTOR in pull-down assays. To further investigate ERK as a possible mediator of S235 phosphorylation, we first developed a phosphospecific antibody that recognized DEPTOR only when it was phosphorylated at S235. Figure 4*A* confirms the specificity of the antibody. WT or S–A mutant DEPTORs were expressed and immunoprecipitated. As shown, the WT DEPTOR was immunodetected with the antibody, whereas the S–A mutant, incapable of S235 phosphorylation, was not.

To test the role of the ERK, we first used ERK inhibitors. As shown in Figure 4*B*, the PD98059 ERK inhibitor (PD) used in the 00 of MG-132 (to prevent DEPTOR degradation) markedly decreased phosphorylation of S235 after a 4 h incubation in 8226 and OPM-2 MM cells. When the PD98059 ERK inhibitor was applied in the absence of MG-132, DEPTOR expression was lost (Fig. 4*C*). Similar results were obtained with the U0126 ERK inhibitor (Fig. S3). The PD98059 ERK inhibitor also decreases binding of DEPTOR to the USP-7 deubiquitinase (Fig. 4*D*), further demonstrating that ERK inhibition phenocopies the effects of drug 3g on DEPTOR. In addition, silencing of ERK1 utilizing two separate shRNAs in either cell line in the presence of MG-132 also inhibited S235 phosphorylation (Fig. 4*E*). In the absence of MG-132, ERK1 knockdown also resulted in loss of DEPTOR expression (Fig. 4*E*, *right panel*). In addition, the PD98059 ERK inhibitor induced dephosphorylation of S235 and loss of DEPTOR expression in primary MM cells from patients (Fig. 4*F*). An *in vitro* kinase assay confirmed the ability of recombinant activated ERK1 to directly phosphorylate DEPTOR on S235 (Fig. 4*G*).

**Figure 5:**
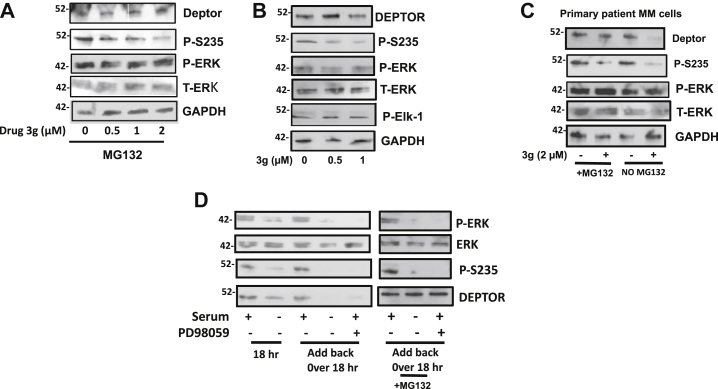
**Regulation of phosphorylation of S235 in MM cells.***A*, 8226 cells exposed to increasing concentrations of drug 3g in the presence of MG132 for 4 h followed by immunoblot assay. *B*, 8226 cells exposed to drug 3g in the absence of MG132 for 4 h followed by immunoblot assay. *C*, primary MM cells exposed to or not exposed to drug 3g in the presence or the absence of MG132 for 6 h followed by immunoblot assay. *D*, MM1.S MM cells cultured with or without serum for 18 h followed by serum add-back for an additional 18 h in the presence or the absence of MG-132. Add-back also in the presence or the absence of PD98059 (50 μM). MM, multiple myeloma; S235, serine 235.

As expected, exposure of 8226 cells to drug 3g in the presence or the absence of MG-132 (Fig. 5, *A* and *B*) also decreased S235 phosphorylation in a concentration-dependent fashion. A similar inhibition of DEPTOR S235 phosphorylation and DEPTOR protein expression was identified in an additional primary MM specimen (Fig. 5*C*). Notably, while preventing DEPTOR S235 phosphorylation, drug 3g had little effect on phosphorylation of ERK or the ERK1 substrate, ELK-1 (Fig. 5, *A*–*C*). Thus, the inhibited ERK-induced phosphorylation of S235 in drug 3g-treated MM cells is not because of a general inhibition of the ERK mitogen-activated protein kinase cascade but, more likely, occurs secondary to a structural alteration of DEPTOR after drug binding.

In contrast to inhibition of ERK1, to test if a stimulation of ERK activity could enhance S235 phosphorylation, we tested effects of serum reconstitution after serum depletion. As the 8226 cell line expresses a mutant rat sarcoma virus (RAS) gene, its ERK activation is not serum dependent. We, thus, performed these experiments in OPM-2 cells and an additional MM cell line with upregulated DEPTOR expression, MM1.S. Figure 5*D* (for MM1.S) and Fig. S4 (for OPM-2) demonstrate that inactivation of ERK (autophosphorylation of ERK) with 18 h of serum starvation inhibits S235 DEPTOR phosphorylation and DEPTOR expression. Continued serum depletion and inhibited ERK over 36 h result in reduced DEPTOR expression, whereas reactivation with serum allows re-expression of DEPTOR. Performing the serum reactivation in the presence of MG-132 (*right side* in Fig. 5*D*) demonstrates that, following reactivation with serum add-back, DEPTOR phosphorylation is reconstituted. Importantly, the rescued effects on ERK reactivation and DEPTOR phosphorylation with serum add-back are prevented by coincubation with the ERK inhibitor, PD98059. Similar results were seen with the OPM-2 cell line (Fig. S4). Thus, using three separate MM cell lines and interventions of ERK inhibition or activation, the results strongly indicate the participation of ERK1 in the phosphorylation of DEPTOR on S235.

### DEPTOR is at the center of ERK–AKT crosstalk in MM cells

**Figure 6:**
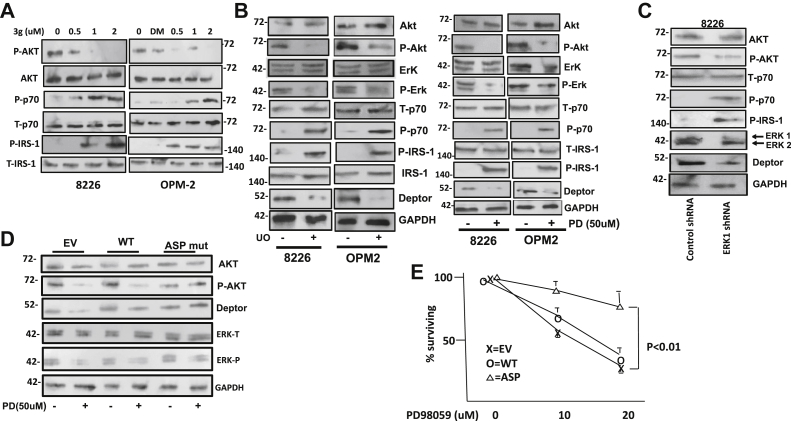
**DEPTOR mediates crosstalk between ERK and AKT.***A*, MM cells exposed to increasing concentrations of drug 3g for 4 h followed by immunoblot assay for AKT phosphorylation (on threonine 308). *B*, MM cells exposed to U0126 or PD98059 ERK inhibitors for 4 h followed by immunoblot assay. *C*, 8226 cells exposed to control or ERK1 shRNA and cultured for 24 h, followed by immunoblot assay. *D*, 8226 MM cells transfected with empty vector, WT DEPTOR, or the phosphomimetic Asp mutant and then challenged in the presence or the absence of PD98059, followed by immunoblot assay. *E*, EV, WT, or Asp mutant–transfected MM cells challenged with PD98059 for 72 h followed by MTT assay. Results are mean ± SD, n = 3. AKT, serine/threonine-specific protein kinase; ASP, aspartate; DEPTOR, Disheveled, Egl-10, and Pleckstrin domain–containing mechanistic target of rapamycin–interacting protein; ERK, extracellular signal–regulated kinase 1; EV, empty vector; MM, multiple myeloma; MTT, 3-(4,5-dimethylthiazol-2-yl)-2,5 diphenyl tetrazolium bromide.

ERK activity in MM cells is crucial for tumor cell proliferation either induced by myeloma growth factors (11, 12) or mutated RAS (13, 14). The critical role for ERK activity in maintaining DEPTOR stability suggested that ERK-induced S235 phosphorylation also played a role in this promyeloma response. This could be explained by DEPTOR-induced effects on AKT. As described previously, there is a negative feedback loop whereby mTORC1/p70 phosphorylation (and subsequent insulin receptor substrate 1 [IRS-1] phosphorylation) inhibits the PI3K–AKT cascade (1). By constraining mTORC1, DEPTOR represses the negative feedback and, thus, promotes AKT phosphorylation/activation. Thus, drug 3g, which prevents DEPTOR–mTOR binding and induces DEPTOR degradation, should reactivate the negative feedback loop. Figure 6*A* demonstrates this effect where drug 3g inhibited AKT phosphorylation in a concentration-dependent fashion in both 8226 and OPM-2 MM cells. We, thus, tested if ERK inhibitors similarly regulated AKT phosphorylation. Lysates from PD98059-treated MM cells (previously shown in Fig. 4*C*) and U0126-treated cells (previously shown in Fig. S4) were reprobed for AKT phosphorylation (on T308). As previously shown, both ERK inhibitors not only induced DEPTOR loss but also significantly downregulated AKT phosphorylation (Fig. 6*B*), consistent with the expected effects of DEPTOR degradation on the negative feedback loop. In similar fashion, shRNA knockdown of ERK1 also inhibited AKT phosphorylation in association with DEPTOR loss (Fig. 6*C*). In these experiments, the targeting of DEPTOR by drug 3g or ERK inhibition concurrently enhanced phosphorylation of p70S6K and IRS-1 (Fig. 6, *A*–*C*), supporting the notion that AKT downregulation was mediated by the negative feedback loop. To confirm that DEPTOR degradation mediated the negative effects of ERK inhibitors on AKT phosphorylation, 8226 MM cells were either transfected with EV, WT DEPTOR, or the phosphomimetic DEPTOR mutant. Transfected cells were then exposed to the PD98059 ERK inhibitor, and effects on AKT were assessed. As shown in Figure 6*D*, PD98059 inhibited AKT phosphorylation in MM cells transfected with an EV or WT DEPTOR, but AKT inhibition was prevented in cells transfected with the phosphomimetic (Asp) version of DEPTOR. Thus, the ERK–AKT crosstalk is mediated by ERK-induced effects on DEPTOR that are regulated by the phosphorylation status of S235. To test if this has ramifications for MM cell viability, MM cells, transfected as in Figure 6*D*, were challenged with the ERK inhibitor. As shown in Figure 6*E*, the cytotoxicity induced by ERK inhibition was prevented specifically by transfection of the phosphomimetic DEPTOR, which remains expressed even when ERK is inhibited. In contrast, MM cells transfected with WT DEPTOR were not protected.

## Discussion

Our investigation on the mechanism by which a small-molecule inhibitor induces DEPTOR degradation has uncovered a novel ERK1-dependent regulation of DEPTOR protein stability. Our results indicate that ERK1-induced phosphorylation of S235 on DEPTOR recruits and/or maintains an interaction with the USP-7 deubiquitinase. USP-7 protects DEPTOR against polyubiquitination and proteasomal degradation. When exposed to the anti-DEPTOR drug 3g, S235 becomes dephosphorylated, USP-7 is released, and polyubiquitination and degradation ensues. Several previous detailed studies (3, 4, 5) elucidated the molecular mechanism of serum-induced DEPTOR degradation indicting mTOR-induced and CK1-induced phosphorylation. Although drug 3g rapidly activates mTOR (presumably by inhibiting DEPTOR:mTOR binding (9)), mTOR-induced phosphorylation is not involved in drug-induced DEPTOR degradation because of the following reasons: (1) drug 3g′s prevention of DEPTOR:mTOR binding would theoretically preclude mTOR-induced phosphorylation of DEPTOR; (2) drug 3g was effective in causing DEPTOR degradation when mTOR was paralyzed by pharmacologic or genetic means (9); and (3) drug 3g induced similar degradation of a DEPTOR mutant in which all residues of the mTOR-specific degron were rendered resistant to mTOR-mediated phosphorylation (9). Our current MS screening for drug 3g-induced PTMs was consistent with these prior results as altered phosphorylation of mTOR-specific and CK1-specific residues was not identified. In contrast, we found a marked reduction in S235 phosphorylation.

Since the first description of the mTOR-dependent regulation of DEPTOR stability, several additional mechanisms have been described. For example, in cardiac myocytes, P38γ and p38δ control heart size by phosphorylation of DEPTOR residues outside the β-TrCP degron with subsequent degradation (15). In contrast, p38α did not alter DEPTOR phosphorylation or protein stability. In data not shown, we found that drug 3g induces a modest activation of p38 kinase activity. However, the p38γ/p38δ-specific phosphorylation events on DEPTOR reported to regulate stability (on S145, S244, S265, S293, and T321) were not identified in screening of drug 3g-treated MM cells. In addition, the expression of p38 in MM cells is specifically the α-isoform (16) as was the activity stimulated by drug 3g. These results strongly argue against a role for p38 in our study. An additional mechanism of DEPTOR turnover was regulated by the KDM4A demethylase which, when inhibited by the 2-hydroxyglutarate oncometabolite in isocitrate dehydrogenase–mutated cells, depressed DEPTOR stability (6). However, it is unclear whether KDM4A plays any role in cells with nonmutated isocitrate dehydrogenase, and we could not identify any drug 3g-induced alteration of DEPTOR methylation. An intriguing mechanism of DEPTOR regulation was also described by Zhao *et al.* (17), which is similar to our results in the critical role of another deubiquitinase, ovarian tumor deubiquitinase, ubiquitin aldehyde binding 1 (OTUB1). In that report, DEPTOR stability was promoted by addition of amino acids to cells, and this was mediated by enhanced binding to OTUB1. The PTMs on DEPTOR that enhanced binding to OTUB1 were not identified but were likely mediated by enzymatic cascades stimulated *via* amino acid supplementation.

Our results support the role for ERK1 activity in maintaining DEPTOR stability and protein expression. As ERK inhibition achieves the identical PTM on DEPTOR as seen with drug 3g (*e.g.*, dephosphorylation of S235), it is not surprising that ERK inhibition also phenocopies the other effects of drug 3g, namely loss of USP-7 binding and DEPTOR degradation. However, the interaction between DEPTOR and ERK may be complex. As mentioned previously, DEPTOR associates with ERK1/2 in pull-down assays (4, 5). However, DEPTOR knockdown results in ERK activation (18), at least in vascular endothelial cells. This suggests a negative feedback loop whereby DEPTOR might be capable of regulating ERK activity. In other words, ERK activation promotes DEPTOR stability/expression, which, in turn, could inhibit ERK activation.

**Figure 7:**
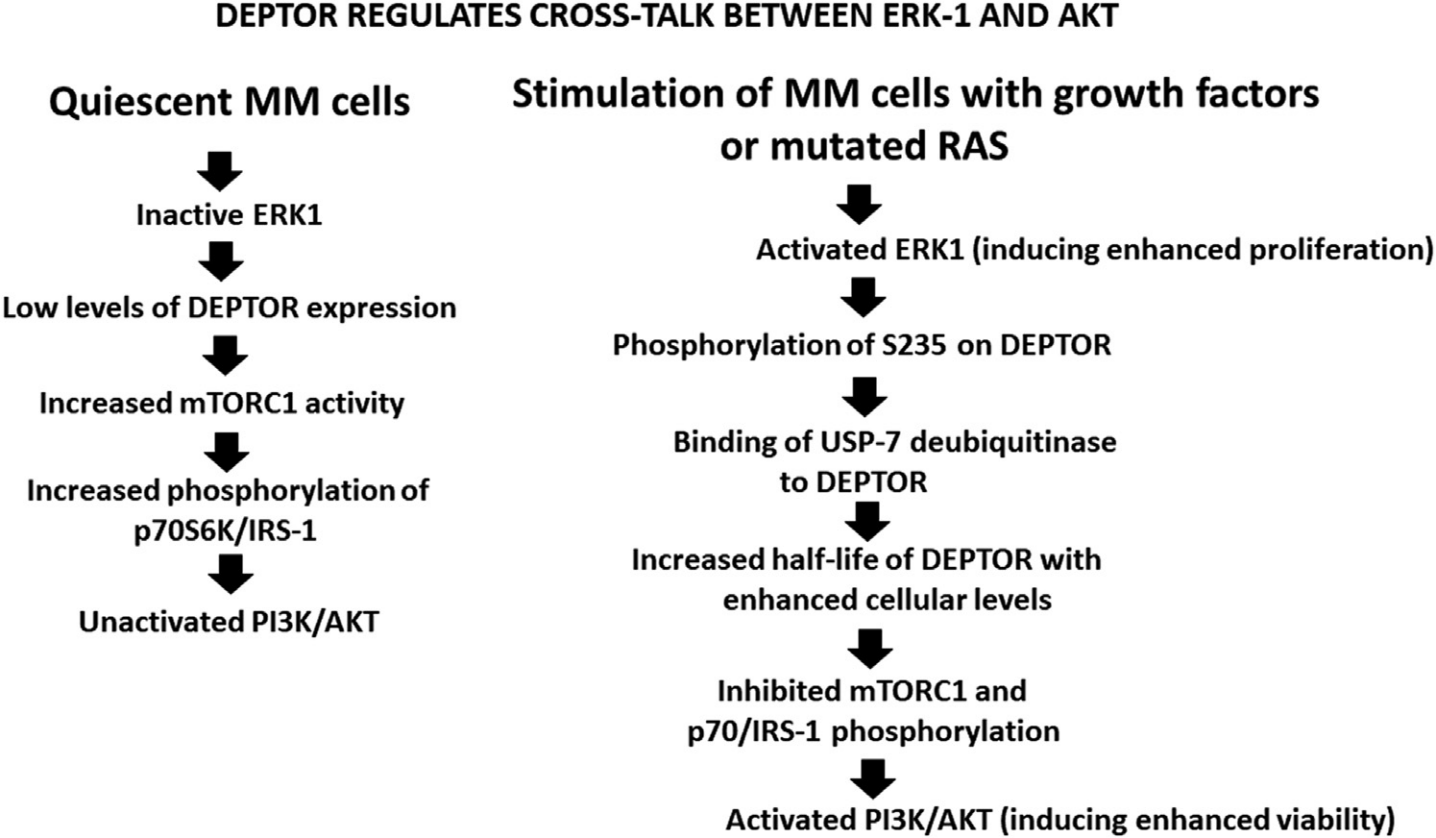
**Hypothesized sequence of events in growth-stimulated myeloma cells pairing ERK1 and AKT activation.** AKT, serine/threonine-specific protein kinase; ERK1, extracellular signal–regulated kinase 1.

Previous work (11, 12, 13, 14) underscores the role of the ERK mitogen-activated protein kinase pathway in stimulating proliferation of MM cells either triggered by MM growth factors (like interleukine-6) or by hyperactive mutated RAS. Our results on the DEPTOR-dependent ERK–AKT crosstalk suggest a mechanism by which MM cell proliferation could be paired with protection of viability as shown in a model of hypothesized events depicted in Figure 7. Through expression of a negative feedback loop, it is well established that the induced inhibition of mTORC1/p70S6K by DEPTOR results in feedback activation of the IRS-1–PI3K–AKT cascade. Thus, as proliferation-promoting ERK activity maintains and prolongs DEPTOR stability, it theoretically combines itself with viability-promoting PI3K–AKT activation. In support of that scenario is the concurrent inhibited AKT phosphorylation when ERK is targeted (Fig. 6, *B* and *C*). Furthermore, our results in Figure 6*D*, demonstrating that only the S235 phosphomimetic DEPTOR version prevented AKT downregulation during ERK targeting, underscores the critical role of S235 DEPTOR phosphorylation in the crosstalk. Indeed, the cytotoxic effect of an ERK inhibitor was significantly protected by ectopic expression of the phosphomimetic DEPTOR (Fig. 6*E*). Thus, myeloma-specific upregulation of DEPTOR expression provides a key step to maintaining cell viability during tumor clonal expansion.

## Experimental procedures

### Cell lines, reagents, and plasmids

The 8226, OPM-2, and MM1.S MM cell lines were obtained from American Type Culture Collection. They were tested for mycoplasma within the last 6 months and were negative. Lentivirus-expressing shRNAs targeting USP-7 were purchased from Origene (catalog no.: TL308454V). The anti-USP-7 drug, P5091, was obtained from Abcam. The ERK inhibitors U0126 and PD98059 were obtained from Millipore Sigma–Aldrich. The phosphospecific antibody recognizing phosphorylated S235 in DEPTOR was generated in rabbits immunized with the phosphorylated peptide Cys-Glu-Leu-Leu-Asn-Glu-Lys-(p-Ser)-Pro-Ser-Ser-Gln-Glu-NH2 (where pSer represents phosphoserine) and subsequently affinity purified. It was specific for phosphorylated peptide *versus* nonphosphorylated peptide when used at dilutions of 1:100 to 1:10,000. It was used in Western blot experiments with dilution at 1:1000. The mutant DEPTOR constructs were generated by cloning full-length complementary DNA of the WT human DEPTOR into plenti6 lentivirus vector as previously described. Mutants with its S235 residue replaced by an alanine or an aspartic acid residue, respectively, were created using Quick-change II Site-directed mutagenesis kit (Agilent Technologies). Lentiviral transduction of MM cells was as described (19).

### Isolation of primary MM cells

Bone marrow aspirate from newly diagnosed patients with MM was separated as previously described (20). Isolated MM cells were >98% pure as shown by microscopy and flow cytometry. This study was approved by the Institutional Review Boards of the Greater Los Angeles VA Medical Center and the UCLA School of Medicine. The study abides by the Declaration of Helsinki principles.

### Evaluation of protein and RNA expression

Binding of endogenous DEPTOR or FLAG-tagged DEPTOR constructs to USP-7 was assayed by coimmunoprecipitation as previously described (9). Real-time PCR analysis for RNA expression and Western blot analysis for protein expression were performed as previously described (20). Pulse-chase assay for turnover of mutant DEPTOR proteins was performed as previously described (9). Polyubuquitination of DEPTOR was evaluated using ubiquitin antibody (catalog no.: 3933) from Cell Signaling.

### Ubiquitination assay

FLAG-DEPTOR-transfected 8226 or OPM-2 MM cells were treated with drug 3g (2 μM) or P5091 (0, 6, or 12 μM) at 37 ^°^C. Cell lysates were immunoprecipitated with FLAG antibody (FLAG immunoprecipitation kit; Millipore Sigma, Inc; catalog no.: FLAGIPT1). Ubiquitination levels of FLAG-DEPTOR were measured with an antiubiquitin antibody used at 1:1000 dilution (Cell Signaling; catalog no.: 58395).

### In-gel digestion for DEPTOR PTM analysis

In-gel digestion was performed as follows: 100 μl of 100 mM ammonium bicarbonate/50% acetonitrile (ACN) was added to gel slices and incubated at 37 °C for 30 min to destain the gel slices. The destaining step was repeated twice until all stain was removed. Next, 50 μl of ACN was added to gel pieces and incubated for 15 min at room temperature to shrink the gel pieces. Gel pieces were dried under speed vac for 5 min, and 50 μl 0.01 mg/ml trypsin solution (dissolved in 100 mM ammonium bicarbonate) was added to gel pieces for digestion at 37 ^°^C overnight. Peptides were extracted from gel bands by 50 μl of 50% ACN/5% formic acid solution for three times, before being dried in speed vac.

### In-solution digestion for identification of proteins binding to DEPTOR

In-solution digestion was performed by first reducing proteins in solution (50 μl) by adding 5 mM Tris(2-carboxyethyl) phosphine at 56 ^°^C for 1 h and then alkylating them by adding 40 mM iodoacetamide at room temperature for 30 min in the dark. Two hundred microliter of cold acetone was then added to the protein solution and stored in −20 ^°^C for 1 h before centrifugation at 13,000*g* for 10 min at 4 ^°^C. The supernatant was then removed, and protein pellets were air dried for 10 min in room temperature. Trypsin dissolved in 50 mM ammonium bicarbonate was added to protein pellets for digestion at 37 ^°^C overnight. Protein digests were desalted by Empore stage-tip the following day. Elution from the stage-tip was dried by speed vac and resuspended in 3% ACN with 0.1% formic acid.

### LC–MS/MS for protein ID, PTM analysis, and label-free quantification

A total of 1.0 μg protein was injected to an ultimate 3000 nano–liquid chromatography (LC), which was equipped with a 75 μm × 2 cm trap column packed with C18 3 μm bulk resins (Acclaim PepMap 100; Thermo Fisher Scientific), and a 75 μm × 15 cm analytical column with C18 2 μm resins (Acclaim PepMap RSLC; Thermo Fisher Scientific). The nano-LC gradient was 3–35% solvent B (A = H_2_O with 0.1% formic acid; B = ACN with 0.1% formic acid) over 40 min and from 35% to 85% solvent B in 5 min at a flow rate of 300 nl/min. The nano-LC was coupled with a Q Exactive Plus orbitrap mass spectrometer (Thermo Fisher Scientific). The electrospray ionization voltage was set at 1.9 kV, and the capillary temperature was set at 275 ^°^C. Full spectra (*m/z* 350–2000) were acquired in profile mode with resolution of 70,000 at *m/z* 200 with an automated gain control target of 3 × 10^6^. The most abundance 15 ions were subjected to fragmentation by higher-energy collisional dissociation with normalized collisional energy of 25. MS/MS spectra were acquired in centroid mode with resolution of 17,500 at *m/z* 200. The automated gain control target for fragment ions are set at 2 × 10^4^ with maximum injection time of 50 ms. Charge states 1, 7, 8, and unassigned were excluded from tandem MS experiments. Dynamic exclusion was set at 45.0 s.

### Data analysis of MS results

Raw data were searched against UniProt human database (released July 2, 2015; number of entries: 23,292) by Proteome Discoverer, version 1.4 (ThermoFisher Scientific) for binding protein identification and label-free quantification. UniProt human DEPTOR sequence (DEPTOR sequence, version 2, released July 7, 2009; number of entries: 1) was used for PTM analysis. “Precursor ions area detector” node is used within Proteome Discoverer, version 1.4, for label-free quantification. Following parameters were set: precursor mass tolerance ±10 ppm, fragment mass tolerance ±0.02 Th for higher-energy collisional dissociation, up to two miscleavages by semitrypsin. Variable modification includes methionine oxidation, lysine methylation, and alkylation; serine, threonine, and tyrosine phosphorylation. Cysteine carbamidomethylation was set as fixed modification. False discovery rate (FDR) was at 1.0% calculated by Proteome Discoverer, version 1.4, percolator node, which searches Decoy database to determine FDR. Minimum of one peptide was required for protein identification.

The phosphorylation percentage was calculated by normalizing the peak area of the phosphorylated peptide to the total peak area of the same peptide (sum of areas from all PTMs and nonmodified form of the same peptide). The quantification information was determined based on two biological replicates, each of which was repeated twice as technical replicates. Student's *t* test was performed to determine the *p* values.

Label-free quantification of DEPTOR-binding proteins was performed based on the average peak areas of top three peptides of each protein. Three biological replicates were used for average to determine the relative abundance of DEPTOR-binding proteins in control (MG-132 alone) and treated (3g + MG-132) samples. *p* Values were calculated based on three biological replicates.

## Data availability

Raw MS data and search results were placed in the MassIVE public repository (https://massive.ucsd.edu/ProteoSAFe/dataset.jsp?task=31938adc56e843aba65dd52304d25c74). Project ID number: MSV000088145. Search engine and release version: “Proteome Discoverer, version 1.4.” Release version/date of sequence database searched: DEPTOR sequence, version 2, released July 7, 2009, human database released, July 2, 2015. Number of entries in the database actually searched human database: number of entries is 23,292; DEPTOR database: number of entries is 1. How FDR was calculated: FDR was calculated by Proteome Discoverer, version 1.4, percolator node, which searches Decoy database to determine FDR.

## Supporting information

This article contains supporting information.

## Conflict of interest

The authors declare they have no conflicts of interest with the contents of this article.
