# Supplementary material for: Turnover of the mTOR inhibitor, DEPTOR, and downstream AKT phosphorylation in multiple myeloma cells, is dependent on ERK1-mediated phosphorylation
Source: J Biol Chem. 2022 Feb 23;298(4):101750. doi: 10.1016/j.jbc.2022.101750 (PMC8933699; doi:10.1016/j.jbc.2022.101750)
Supplement: Supplemental Figures S1–S4 and Tables S1–S2 [file mmc1.docx]

TITLE: Turnover of the mTOR inhibitor, DEPTOR, and downstream AKT phosphorylation in multiple myeloma cells, is dependent on ERK1-mediated phosphorylation

Authors: Mario Vega, Yi Chen, Yijiang Shi, Joseph Gera, Alan Lichtenstein

Material included:

In Word doc- Supporting Figure S1

Supporting Figure S2

Supporting Table S1

Supporting Table S2

Supporting Figure S3

Supporting Figure S4

Supporting fig 1:MS/MS spectrum of peptide "LMELLNEKSPSSQETHDSPFCLR" with S235 phosphorylation

Intensity [counts](10E3)

500 1000 1500

m/z

0 50 100 150 200

**Supporting figure 2: A)** Immunoblot assay for expression of wild type or S-to-Alanine mutant (ALA) 6 hrs after transfection when cultured with MG132 and +/- drug 3g. **B)** Pulse-chase assay assessing turn-over and T_1/2_  of wild type DEPTOR (WT) versus phosphomimetic S-ASP mutant.

|  |  |  |
| --- | --- | --- |
| **ACCESSION** | **DESCRIPTION** | **P VALUE (two-tailed)** |
| Q13501 | Sequestosome-1 OS | 0.002 |
| P11441 | Ubiquitin-like protein 4A | 0.012 |
| P18085 | ADP-ribosylation factor 4 | 0.031 |
| Q9UNM6 | 26s proteasome non-ATPase regulatory subunit B | 0.006 |
| Q16891 | MICOS complex subunit MIC60 | 0.002 |
| P17612 | cAMP-dependent protein kinase catalytic subunit alpha | 0.04 |
| O95168 | NADH dehydrogenase 1 beta subcomplex subunit 4 | 0.001 |
| Q16204 | Coiled-coil domain-containing protein 6 | 0.009 |
| P46379 | Large proline-rich protein BAG 6 | 0.003 |
| O00159 | Unconventional myosin-1c | 0.003 |
| Q9POJO | NADH dehydrogenase 1 alpha subcomplex subunit | 0.017 |
| P42345 | mTOR | 0.02 |
| Q9UNF1 | Melanoma-associated antigen D2 | 0.009 |
| O00148 | ATP-dependent RNA helicase DDX39A | 0.03 |
| P47929 | Galactin-7 | 0.002 |
| Q14145 | Kelch-like ECH-assocprotein 1 | 0.0003 |
| P09543 | 2’3’-cyclic nucleotide 3’phosphodiesterase | 0.002 |
| P13861 | c-AMP-dep protein kinase type II-a regulatory subunit | 0.028 |
| Q969X5 | ER-Golgi intermediate compartment protein 1 | 0.004 |
| Q9UKV8 | Protein argonaute-2 OS | 0.01 |
| Q9Y678 | Coatomer subunit gamma-1 | 0.012 |
| P02786 | Transferrin receptor protein 1 | 0.00004 |
| P31930 | Cytochrome b-c1complex subunit 1, mitochondrial | 0.03 |
| Q93009 | Ubiquitin carboxyl-terminal hydrolase 7 OS | 0.000001 |
| O60313 | Dynamin-like 120kDa protein, mitochondrial | 0.002 |
| Q9P1W9 | PIM-2 kinase | 0.04 |
| Q9PUBQ5 | eIF3 subunit K | 0.02 |
| P41240 | Tyrosine protein kinase CSK 05 | 0.008 |
| Q7Z2W4 | Zinc finger CCCH-type antiviral protein 1 | 0.001 |
| P98171 | Rho-GTPase activating protein 4 | 0.02 |
| O95071 | E3 ubiquitin ligase UBR5 | 0.016 |
| O00139 | Kinesin-like protein KIF2A | 0.0003 |
| O00767 | Acyl-CoA desaturase OS | 0.002 |
| Q13751 | Laminin subunit beta-3 | 0.003 |
| Q12824 | SWI/SNF-related matrix assoc actin-dependent regulator | 0.001 |
| Q5WcB1 | Ring finger protein 219 | 0.04 |
| Q8IW41 | MAPkinase activated protein 5 | 0.005 |
| Q9UMZ2 | Synergin gamma OS | 0.003 |
| Q9NZ08 | ER aminopeptidase 1 | 0.0007 |
| Q05086 | Ubiquitin protein ligase E3A | 0.005 |
| Q9BV68 | E3 ubiquitin ligase RNF126 | 0.04 |
| Q94679 | Ancient ubiquitous protein 1 | 0.05 |
| Q9UKA4 | A-kinase anchor protein 11 | 0.003 |
| Q9NR50 | Translation initiation factor eIF-2B subunit gamma | 0.005 |
| Q9Y282 | ER-Golgi intermediate compartment protein 3 | 0.004 |
| P62714 | Phosphatase 2A catalytic subunit | 0.0004 |
| O75592 | E3 ubiquitin ligase MYCBP2 | 0.05 |
| Q99700 | Ataxin 2 | 0.012 |
| Q5TAQ9 | DDBI- and CUL4 associated factor 8 | 0.005 |
|  |  |  |

Supporting Table 1: List of proteins whose binding to DEPTOR is significantly decreased following exposure to drug 3g.

| **ACCESSION** | **Description** | **P value** |
| --- | --- | --- |
| Q06323 | Proteasome activation complex subunit 1 | 0.03 |
| P49720 | Proteasome subunit beta type-3 | 0.027 |
| P62312 | U6 snRNA-assoc Sm-like protein LSm6 | 0.004 |
| P28065 | Proteasome subunit beta type 9 | 0.039 |
| Q15126 | Phosphomevalonate kinase | 0.005 |
| P26447 | Protein S11-A4 | 0.003 |
| Q96C19 | EF hand domain-containing protein D2 | 0.002 |
| Q9BZE9 | Tether containing UBXdomain for GLUT4 | 0.04 |
| O15143 | Actin-related protein 2/3 complex subunit 1B | 0.005 |
| P61163 | Alpha-centractin | 0.006 |
| P35606 | Coatomer subunit beta | 0.03 |
| O15145 | Actin-related protein 2/3 complex subunit 3 | 0.005 |
| Q92890 | Ubiquitin fusion degradation protein 1 | 0.05 |
| O43920 | NADH dehydrogenase iron-sulfur protein 5 | 0.05 |
| Q8N5C8 | TGF-beta-activated kinase 1 and MAP3K7-binding protein 3 | 0.03 |
| Q99436 | Proteasome subunit beta type 7 | 0.035 |
| P05783 | Keratin type 1 cytoskeletal 18 | 0.015 |
| Q01813 | ATP-dependent 6-phosphofructokinase- platelet type | 0.02 |
| P54578 | Ubiquitin C-terminal hydrolase 14 | 0.05 |
| Q8WXX5 | DnaJ homolog subfamily C member 9 | 0.004 |
| O14773 | Tripeptidyl-peptidase 1 | 0.005 |
| Q9GZR7 | ATP-dependent RNA helicase DDX24 | 0.002 |
| P28062 | Proteasome subunit beta type 8 | 0.003 |
| Q9BRP4 | Proteasome ATPase-associated factor 1 | 0.002 |
| P28065 | Proteasome subunit beta type 9 | 0.05 |

Supporting Table 2: List of proteins whose binding to DEPTOR is significantly increased following exposure to drug 3g

Supporting figure 3: MM cells exposed to the U0126 ERK inhibitor for 4 hours in the presence or absence of MG-132, followed by immunoblot assay. The Erk, P-Erk, Deptor and GAPDH images in 8226 and OPM-2 cells treated with UO126 but without MG-132 (left sided images) were also used in figure 6B where the effect of the UO126 inhibitor on AKT phosphorylation was demonstrated.

Supporting fig 4: OPM-2 cells cultured +/- serum overnight (18 hrs)

with aliquots taken for immunoblot assay; Remaining cells re-cultured

for an additional 18 hrs +/- serum and +/- PD98059, followed by

immunoblot assay.
